# Inter- and intra-specific variation in hair cortisol concentrations of Neotropical bats

**DOI:** 10.1093/conphys/coab053

**Published:** 2021-07-14

**Authors:** Natalia I Sandoval-Herrera, Gabriela F Mastromonaco, Daniel J Becker, Nancy B Simmons, Kenneth C Welch

**Affiliations:** 1 Department of Ecology and Evolutionary Biology, University of Toronto, Ontario, M5S 3B2, Canada; 2 Department of Biological Sciences, University of Toronto Scarborough, Ontario, M1C 1A4, Canada; 3 Reproductive Sciences, Toronto Zoo, Ontario, M1B 5K7, Canada; 4 Department of Biology, University of Oklahoma, Norman, OK 73019, USA; 5 Department of Mammalogy, Division of Vertebrate Zoology, American Museum of Natural History, New York, NY, 10024-5102, USA

## Abstract

Quantifying hair cortisol has become popular in wildlife ecology for its practical advantages for evaluating stress. Before hair cortisol levels can be reliably interpreted, however, it is key to first understand the intrinsic factors explaining intra- and inter-specific variation. Bats are an ecologically diverse group of mammals that allow studying such variation. Given that many bat species are threatened or have declining populations in parts of their range, minimally invasive tools for monitoring colony health and identifying cryptic stressors are needed to efficiently direct conservation efforts. Here we describe intra- and inter-specific sources of variation in hair cortisol levels in 18 Neotropical bat species from Belize and Mexico. We found that fecundity is an important ecological trait explaining inter-specific variation in bat hair cortisol. Other ecological variables such as colony size, roost durability and basal metabolic rate did not explain hair cortisol variation among species. At the individual level, females exhibited higher hair cortisol levels than males and the effect of body mass varied among species. Overall, our findings help validate and accurately apply hair cortisol as a monitoring tool in free-ranging bats.

## Introduction

Free-living animals face multiple natural and anthropogenic challenges that threaten their survival and thus are of considerable interest to ecophysiologists concerned with the study of effects of stress on vertebrates. One of the most extensively studied processes associated with response to stressors (biotic or abiotic environmental factors that disrupt homeostasis; Schulte, 2014) is the release of glucocorticoid (GC) hormones (Creagh and Brendan Delehanty, 2013; MacDougall-Shackleton *et al.*, 2019). GCs are known to facilitate the mobilization of energy required to cope with stressors and, during normal conditions, play a key role in regulating growth, circadian activity and energy metabolism (review in Landys *et al.*, 2006). Levels of GCs are commonly employed as a biomarker of allostatic load or stress (indirect indicators of health) (Sapolsky *et al.*, 2000; Wikelski and Cooke, 2006; Pearson Murphy, 2007; Busch and Hayward, 2009). GC secretion is a well-conserved process across vertebrates and involves activation of the hypothalamic–pituitary–adrenal (HPA) axis and release of GCs from the adrenal glands to the blood stream (Norris and Carr, 2013). In mammals, the primary GC is cortisol, which induces a cascade of events to maintain homeostasis at multiple target tissues (Pearson Murphy, 2007; Boonstra, 2013). An acute increase in GC levels can benefit an individual’s survival (e.g. by allocating energy in defence and escape) yet if adverse conditions remain, continuously elevated GCs in circulation can become pathological, causing immune suppression, neuronal cell death and reproductive impairment (Sapolsky *et al.*, 2000; Tilbrook, 2000; Wingfield and Romero, 2011; Hing *et al.*, 2016).

Although many of the environmental challenges that wild populations experience are chronic (e.g. prolonged food deprivation, climate change, habitat disturbance, pollution), studies of stress physiology have focused on detecting acute stress by looking at GC levels in blood, urine and faeces (Sheriff *et al.*, 2011; Creagh and Brendan Delehanty, 2013). The rapid turnover of these tissues, however, only gives short-term information of HPA activity over periods of hours or days (Sheriff *et al.*, 2011), which may not be an appropriate time scale. Assessment of cortisol in tissues with slower turnover rates, such as hair, may reflect circulating cortisol levels over longer periods of several weeks or even months, which is the time scale over which chronic environmentally induced stress would be expected to occur (Davenport *et al.*, 2006; Macbeth *et al.*, 2010; Ashley *et al.*, 2011; Mastromonaco *et al.*, 2014). Cortisol is incorporated into developing hairs from the blood stream during periods of active hair growth, allowing researchers to retrospectively examine cortisol production at the time that a stressor or stressors were faced (Davenport *et al.*, 2006; Pragst and Balikova, 2006). Hair can be collected in a minimally invasive manner, is usually easily accessible in relatively large amounts and is easy to store and transport, all of which make it particularly useful for wildlife studies, especially those involving threatened or endangered species (Koren *et al.*, 2002; Macbeth *et al.*, 2010, 2012). Hair cortisol levels are not likely affected by stress induced by capture and/or handling, which is one of the main limitations of blood GC analysis (Russell *et al.*, 2012). A single sample of hair can also provide complementary and valuable information about ecology and behaviour, including diet and movement (e.g. using stable isotope analyses; Fraser *et al.*, 2010; Sullivan *et al.*, 2012; Voigt *et al.*, 2012; Oelbaum *et al.*, 2019), condition (e.g. nutrition; Montillo *et al.*, 2019), toxicant exposure (Hernout *et al.*, 2016; Becker *et al.*, 2018) and molecular identification (Magioli *et al.*, 2019), opening possibilities for more integrative studies. However, analyses of hair samples can be challenging. Despite being a very promising tool for assessing wildlife health, quantifying hair cortisol is a method that has limitations; although these are largely based on lack of detailed knowledge of patterns of hair growth (Meyer and Novak, 2012; Russell *et al.*, 2012; Sharpley *et al.*, 2012). For example, the exact time scale reflected in any given sample will depend on the rate of hair growth and moulting patterns; this information is unknown for most species, which makes the time window being evaluated unclear (Koren *et al.*, 2002; Fourie *et al.*, 2016). Moreover, rates of cortisol incorporation to the hair shaft are known to differ across body regions and among species (Sharpley *et al.*, 2012; Acker *et al.*, 2018; Lavergne *et al.*, 2020). Nevertheless, hair cortisol levels offer a potentially powerful tool for assessing relatively long-term stress levels in mammals.

Hair cortisol and its correlation with natural and anthropogenic stressors has been explored for different wild mammals, including rhesus monkeys (*Macaca mulatta*; Dettmer *et al.*, 2014), grizzly bears (*Ursus arctos*; Macbeth *et al*., 2010), reindeer/caribou (*Rangifer tarandus*; Ashley *et al*., 2011), lynx (*Lynx canadensis*; Terwissen *et al.*, 2013; Azevedo *et al.*, 2020), mongoose *(Herpestes ichneumon*; Azevedo *et al.*, 2019) and snowshoe hares (*Lepus americanus*; Lavergne *et al.*, 2020); other examples reviewed by Kalliokoski *et al.*, 2019). Although most of these studies support hair cortisol as an informative measure of central HPA activity, they also identified intrinsic factors such as age, sex, reproductive stage and social status that modulate GC levels in different contexts (Wingfield and Romero, 2011; Crespi *et al.*, 2013; Hau *et al.*, 2016). Not accounting for these intrinsic sources of variation in GC levels may lead to incorrect or misleading estimates of the effects of stressors on individual fitness and population health (Sapolsky *et al.*, 2000; Reeder and Kramer, 2005; Busch and Hayward, 2009; Wingfield and Romero, 2011; Kalliokoski *et al.*, 2019).

Ecological traits such as diet, fecundity and lifespan, as well as phylogenetic relatedness, have been proposed to explain differences in baseline cortisol levels in wild species (Wingfield and Romero, 2011; Patterson *et al.*, 2014). Evolution of different life-history strategies are also thought to have led to different adaptations in HPA activity modulation so as to maximize individual fitness within species (Bonier *et al.*, 2009; Bonier and Martin, 2016). Bats are a very ecologically diverse group comprising over 1400 species that live in most terrestrial ecosystems and have a wide variety of diets, use many different roost types and have many different social systems (Kunz and Fenton, 2005; Dumont *et al.*, 2012; Gunnell and Simmons, 2012; Simmons and Cirranello, 2020). This diversity provides the opportunity to study the ecological correlates of cortisol levels among phylogenetically related species with different life history traits. Few ecological correlates of GCs have been evaluated simultaneously in mammalian groups in the context of cortisol studies, and fewer studies have further related cortisol levels to life history traits across multiple species from a single mammalian clade. Among bats, variation in plasma cortisol levels associated with seasonal food availability has been studied in two species with contrasting diets, *Carollia perspicillata* and *Desmodus rotundus* (Lewanzik *et al.*, 2012), but no other comparative studies have been conducted within this order. Furthermore, little is known about the modulation of the stress response in bats, despite Chiroptera being the second-most speciose order of mammals.

Bat populations are declining worldwide due to ongoing habitat destruction and land use changes, increased interaction with human environments and associated threats including wind turbine fatalities, hunting and targeted killing, pesticide exposure and emerging infectious diseases such as white-nose syndrome (O’Donnell, 2000; Mickleburgh *et al*., 2002; Kunz *et al*., 2007; Frick *et al*., 2010; Racey, 2013; Voigt and Kingston, 2015). Because many bat species are threatened or have declining populations in parts of their range (IUCN Red List of Threatened Species, 2020), minimally invasive tools to monitor colony health and identify cryptic stressors are critically needed to efficiently direct conservation efforts. It is essential to investigate the factors influencing baseline GCs to properly detect elevated cortisol levels due to long-term stressors.

In this study, we describe intra- and inter-specific sources of variation in baseline hair cortisol levels in bats, which contributes to better understanding the potential for hair cortisol to be an indicator of HPA activity in this taxon. We hypothesize that interspecific variation in hair cortisol of bats will be greater than intra-specific variation and that such heterogeneity will be best explained by ecological traits directly related to energy expenditure, such as basal metabolic rate (BMR), dietary guild, foraging style and roost durability. We expect that species with high energetic demands or less predictable energy acquisition (e.g. less reliable food sources) will have higher hair cortisol. Specifically, we predict the following: (i) a positive relationship between BMR and hair cortisol;(ii) bats that feed on fruit and nectar, which are energy rich and readily available, will have lower hair cortisol; (iii) bats that actively hunt prey during flight, such aerial hawkers, will have higher GC levels owing to greater energetic demands compared to gleaners that can hunt from perches (Norberg and Rayner, 1987; Fenton, 1990); and (iv) species using more ephemeral day roosts (e.g. foliage or crevices under exfoliating bark) will have higher hair cortisol than species using more stable structures (Kunz and Fenton, 2005).

**Figure 1:**
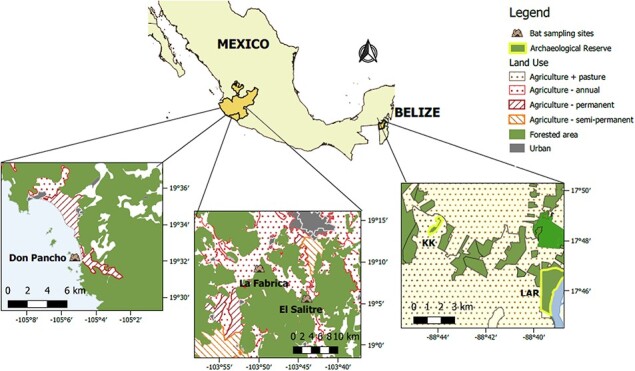
Sampling sites in central Belize and Mexico showing the use of land in the surrounding areas. Sources: Sistema Nacional de Información Estadística y Geográfica de Mexico (INEGI, 2013) and Biodiversity and Environmental Resource Data System for Belize.

## Material and methods

### Study sites

We sampled bats from northern Belize (Orange Walk District) and two locations in Mexico (Colima and Chihuahua States). In each region, we sampled sites with different levels of habitat fragmentation and agricultural intensity. We used the global Human Modification Index (HMI; Kennedy *et al.*, 2019) as a standardized measure of disturbance, using a 5-km buffer around each collection site. The HMI is a cumulative measurement with possible values between 0 (no disturbance) and 1 (highest disturbance) that includes transportation, human settlement, agriculture, extractive activities and electric infrastructure (Kennedy *et al.*, 2019). Sites were classified as low (0 ≤ HMI ≤ 0.10), moderate (0.10 < HMI ≤ 0.40), high (0.40 < HMI ≤ 0.70) and very high (0.70 < median HMI ≤ 1.00). At all sites, bats were captured from 18:00 to 22:00 using mist nets and from 18:00 to 5:00 using harp traps (only in Belize) set along flight paths. Bats sampled during the day were captured in their roosts, mainly caves, using hand nets. We recorded sex, size (body mass, g) and reproductive stage (females: pregnant, lactating; males: active, inactive; Kunz and Parsons, 2009). Reproductive stage was assessed by checking for the presence of scrotal testes in males (indicating that the individual was reproductively active at the time of capture) and by the evidence of pregnancy or lactation (enlarge nipples) in females (Racey, 2009). Only adult individuals were sampled for hair collection.

In Colima (west central Mexico) in March 2019 (dry season), we sampled bats roosting in three caves surrounded by different levels of disturbance: Don Pancho Cave (moderate disturbance, HMI = 0.38), El Salitre Cave (high disturbance, HMI = 0.44) and La Fábrica Cave (high disturbance, HMI = 0.57; Fig. 1). Don Pancho Cave is located on San Agustin island, 1 km away from the coast of Chamela Bay, Jalisco (19.5353°N, −105.0881°W). El Salitre Cave is near Los Ortices village, Colima (19.083330°N, −103.726667°E). La Fábrica Cave is 6.4 km southwest of Coquimatlan town, Colima (19.1513°N, −103.8353°W). We refer here to these locations collectively as central Mexico, and we took samples from three species in this region: *Leptonycteris yerbabuenae*, *Macrotus waterhosii*, and *Pteronotus mexicanus*. We also sampled bats foraging close to pecan nut croplands near the town of Jimenez, Chihuahua (northern Mexico). This region is entirely dedicated to the production of pecan nuts with thousands of squared kilometres of cultivated land (Orona Castillo *et al.*, 2018). We visited one that farms using organic practices and another that farms with intensive use of pesticides. However, the estimated HMI was the same for the two sites (HMI = 0.49, high disturbance). We collected hair samples from three bat species (*Antrozous pallidus*, *Tadarida brasilensis* and *Myotis velifer*) at both northern Mexico sites.

Our field sites in Belize consisted of two forest patches of very different size located ~10 km apart and separated by a heterogeneous, largely agricultural landscape. Lamanai Archaeological Reserve (LAR) is a protected secondary semi-deciduous forest of 450 ha with a high canopy and with relatively low disturbance (HMI = 0.17) (Herrera *et al.*, 2018). In contrast, the Ka’Kabish archaeological site (KK) is a small remnant forest patch of ~45 ha surrounded by cattle pastures and local croplands (Fig. 1). Although the landscape in Belize is apparently disturbed and highly fragmented, agricultural activity and urban development is not as intense as the field sites in Mexico, which is reflected in their moderate HMI scores (LAR: 0.17; KK: 0.18). We collected hair samples from 12 different species (Table 1) in April 2018 and 2019 (dry season) at Belize sites.

### Ethical statement

Field procedures followed guidelines for safe and humane handling of bats published by of the American Society of Mammalogists (Sikes and Bryan, 2016) and were approved by the Institutional Animal Care and Use Committees of the University of Georgia (A2014 04-016-Y3-A5), University of Toronto (20012113) and American Museum of Natural History (AMNHIACUC-20180123). Fieldwork was authorized by the Belize Forest Department under permits WL/2/1/18(16) and WL/1/19(06). Sample collection in Mexico was approved under the permit #FAUT-0069.

### Sample collection

We manually trimmed a hair sample (3–10 mg) using round tip curved dissection scissors from the scapular region on the back of each bat. The resulting samples were placed individually in 1–2-ml plastic tubes using flat tweezers. The dissection tools were cleaned with 70% ethanol between sampling different individuals to avoid cross contamination. The amount of hair removed from each bat depended on the hair density of each species. The hair shaft was carefully cut close to the root avoiding removing skin or follicle tissue. From pilot analyses, we determined a minimum amount of 3 mg of hair was necessary to obtain values around 50% binding on the standard curve thereby accurately estimating cortisol concentration in the sample.

**Table 1:** Species-level ecological traits and hair cortisol data for 18 Neotropical bat species

Family	Species	Region	*N*	Mass(g)	Dietary guild	Foraging style	WAR	Roost durability	Fecundity (litter/yr)	Colony size	Lifespan (years)	Cortisol (ng/g)		
				Mean ± SD								Mean	±	SD
Emballonuridae	*R. naso*	*BZ*	14	4.54 ± 1.47	Animalivory	Aerial forager	6.5	2	>1	Small	5	639.57	±	392.10
	*Saccopterix billineata*	*BZ*	20	6.79 ± 1.12	Animalivory	Aerial forager	6.1	2.4	1	Small	5	347.28	±	232.01
Molossidae	*M. nigricans*	*BZ*	5	35.00 ± 2.35	Animalivory	Aerial forager	11.1	4	1	Large	10	227.80	±	123.54
	*T. brasilensis*	*NMX*	23	10.73 ± 0.63	Animalivory	Aerial forager	8.6	5.3	1	Large	10	1782.87	±	751.07
Mormoopidae	*P. mesoamericanus*	*BZ*	26	17.08 ± 2.06	Animalivory	Aerial forager	6.7	5.6	1	Large	10	218.79	±	76.48
	*P. mexicanus*	*CMX*	35	13.37 ± 1.18	Animalivory	Aerial forager	6.7	5.6	1	Large	10	1246.96	±	1663.56
Phyllostomidae	*D. rotundus*	*BZ*	22	28.01 ± 3.54	Animalivory	Gleaner	6.7	4.5	>1	Large	15	395.00	±	226.92
	*G. soricina*	*BZ*	19	10.33 ± 1.87	Phytophagy	Gleaner	6.4	4.6	>1	Medium	10	99.41	±	117.86
	*L. yerbabuenae*	*CMX*	18	22.26 ± 1.72	Phytophagy	Gleaner	7	5.6	1	Large	15	24614.50	±	14780.52
	*Lophostoma evotis*	*BZ*	1	19.00±	Animalivory	Gleaner	5.3	3	1	Small	20	3046.00	±	
	*M. waterhousii*	*CMX*	23	14.31 ± 2.63	Animalivory	Gleaner	5.8	5.6	1	Medium	10	616.37	±	415.85
	*Mimon cozumelae*	*BZ*	3	25.00 ± 0	Animalivory	Gleaner	8.3	2.8	1	Small	20	2000.67	±	1284.91
	*S. parvidens*	*BZ*	17	14.61 ± 1.55	Phytophagy	Gleaner	6.5	4	>1	Small	20	635.97	±	379.79
	*Trachops cirrhosus*	*BZ*	3	31.50 ± 0.50	Animalivory	Gleaner	6.3	3.9	1	Medium	10	147.10	±	22.36
Vespertilionidae	*A. pallidus*	*NMX*	10	15.80 ± 1.70	Animalivory	Gleaner	6.5	4	>1	Small	10	42.79	±	17.46
	*E. furinalis*	*BZ*	7	7.57 ± 0.45	Animalivory	Aerial forager	6.2	3	>1	Large	20	36.66	±	40.54
	*Lasiurus ega*	*BZ*	2	12.00 ± 4.24	Animalivory	Aerial forager	7.9	2.5	>1	Small	15	142.83	±	18.14
	*M. velifer*	*NMX*	11	8.98 ± 1.02	Animalivory	Aerial forager	6.7	2.5	1	Large	10	211.65	±	170.79

Capture sites: BZ, Belize; NMX, Northern Mexico; CMX, Central Mexico. N, number of hair sample analysed for cortisol. WAR, Wing Aspect Rati.

Species were assigned to two dietary guilds: animalivory (insectivorous, sanguivorous and carnivorous) and phytophagy (frugivorous and nectarivorous species). Roost category assignment followed Patterson *et al*., 2007.

### Extraction and quantification of cortisol

Hair samples were processed and analysed at the endocrinology laboratory at the Toronto Zoo following methods described by Acker *et al.*, 2018. Each hair sample was spread apart and weighed in a 7-ml glass scintillation vial. To avoid contamination with other biological fluids, all hair samples were washed with 100% methanol by vortexing in a tube for 10 s and immediately removing the methanol using a pipettor. Immediately thereafter, 100% methanol was added to each sample, at a ratio of 0.005 g/ml. Samples were then mixed for 24 hrs on a plate shaker (MBI Orbital Shaker; Montreal Biotechnologies Inc., Montreal, Quebec City, Canada). After 24 hrs the vials were centrifuged for 10 min at 2400 g. The supernatants were pipetted off into clean glass vials and dried down under air in a fume hood. The dried extracts were stored at −20°C until analysis.

Samples were brought to room temperature prior to analysis. Reconstitution of the desiccated extracts was done by adding phosphate buffer and vortexing for 10 s. Belize samples were reconstituted neat (i.e. evaporated 150 ul and reconstituted with 150 ul), and Mexico samples were reconstituted as follows: three species were neat, two species diluted 1:5 and one species diluted 1:50 in phosphate buffer (Andreasson *et al.*, 2015). Cortisol concentrations were determined using an enzyme immunoassay (EIA) previously described (Dulude-de Broin *et al.*, 2019) but antibody dilution was adjusted. Antibody (R4866, C. Munro, University of California, Davis) and horseradish peroxidase dilutions were 1:10200 and 1:33400, respectively. Cortisol, rather than corticosterone, was targeted because it has been found to be the primary circulating GC in bats, with concentrations four times higher than corticosterone (reviewed in Kwiecinski and Damassa, 2000). Biochemical validation (parallelism and recovery) of the cortisol EIA was done using pooled hair extracts (see Supplementary data). We used pooled hair samples from Big Brown bats (*Eptesicus fuscus*) from a captive colony at McMaster University as a model for species with low cortisol concentrations (i.e. extracts reconstituted neat). For species with diluted extracts (*Tadarida brasiliensis*, *P. mexicanus* and *L. yerbabuenae*) there was sufficient hair for separate pools. The inter-assay coefficient of variation (CV) for high control (24% binding) and low control (60% binding) were 9.3% and 9.8%, respectively. The intra-assay CV was 8.5%. The limits of detection and quantitation were 56 pg/ml and 153 pg/ml, respectively. Results are presented as nanograms of cortisol per gram of hair.

### Validation of immunoassay

Biochemical validations showed that the cortisol assay was suitable for hair. The recoveries of known concentrations of exogenous cortisol from hair extracts were 87.1 ± 3.5% (dilution neat, *E. fuscus*), 98.8 ± 6.3% (dilution, 1:5; *T. brasiliensis*), 105.4 ± 4.7% (dilution, 1:5; *P. mexicanus*) and 101.3 ± 5.2% (dilution, 1:50; *L. yerbabuenae*). The measured hormone concentrations in the spiked samples correlated with the expected concentrations (*E. fuscus*: *r* = 0.997, *P* < 0.001; *T. brasiliensis*: *r* = 0.982, *P* < 0.01; *P. mexicanus*: *r* = 0.999, *P* < 0.001; *L. yerbabuenae*: *r* = 0.995, *P* < 0.001; Supplementary data). Serial dilutions of pooled hair extracts showed parallel displacement with the cortisol standard curve (*E. fuscus*: *r* = 0.991, *P* < 0.01; *T. brasiliensis*: *r* = 0.988, *P* < 0.01; *P. mexicanus*: *r* = 0.997, *P* < 0.001; *L. yerbabuenae*: *r* = 0.996, *P* < 0.001; Supplementary data).

### Species ecological traits

We compiled data on ecological traits considered relevant to cortisol mobilization from previously published literature and databases. Values for traits are species-level averages and may not reflect specific values at these sites (Table 1). Data on BMR was extracted from the literature (Cruz-Neto *et al.*, 2001; Genoud *et al.*, 2018; see Supplementary data) and when not available (*n* = 2) the following formula was used for the estimation: $\ln BMR=0.744\times \ln mass( in\ g)+1.0895$ (Speakman and Thomas, 2003). Information on diet, foraging style, percentage of invertebrates in the diet and fecundity was extracted from the Elton Traits, PanTHERIA and Amniote Life History databases (Jones *et al.*, 2009b; Wilman *et al.*, 2014; Myhrvold *et al.*, 2015). We collapsed variation in diet into two dietary guilds: phytophagy (including nectarivores and frugivores) and animalivory (insectivores and carnivores) because many bat species in our study have diets that combine more than one food source within these categories (Fenton *et al.*, 2001; Kunz and Fenton, 2005; Reid, 2009; Oelbaum *et al.*, 2019). We also considered the percentage of invertebrates in the diet of the animalivorous bats, which can vary significantly among species. Because foraging behaviour is a complex and plastic trait, we simplified this variable into two categories: aerial foragers (i.e. hawkers) and gleaners (including species that glean plant products like fruit as well as insects) since these behaviours may reflect differences in energetic demands associated with foraging (Herrera *et al.*, 2018). Because wing morphology can strongly influence the energetic costs of flight, we also included the mean wing aspect ratio for each species (Norberg and Rayner, 1987; Bullen *et al.*, 2014). Fecundity was defined as the annual average fecundity (litter size × number of litters per year). We estimated roost durability following the methods of Patterson *et al.* (2007), where 1 indicates the most ephemeral and least protected roost types (e.g. rolled leaves and foliage) and 6 indicates the most permanent and protected roost types (e.g. caves). For species known to multiple use different kinds of roost, intermediate ranks were calculated, weighting roost categories according to the relative frequency of use reported in the literature (Schneeberger *et al.*, 2013). Lifespan was drawn from the Animal Ageing and Longevity database (AnAge: The Animal Ageing and Longevity Database, 2020) and DATLife (DATLife Database, 2020). Lifespan was grouped in five categories: 0–5, 5–10, 10–15, 15–20 and >20 years. For many of the species in these databases, longevity estimates are based on captive animals, which likely overestimates life expectancy in the wild. Because bats of a single species may live in colonies of varying sizes, and most values of colony size are reported in ranges in the literature, we classified maximum colony sizes reported for each species as small (1–50), medium (50–500) or large (>500) *sensu*  Santana *et al.* (2011).

### Data analysis

We first used phylogenetic generalized least squares (PGLS) models to evaluate the effect of species-level ecological variables on hair cortisol concentrations while accounting for bat phylogenetic relatedness. We used the *rotl* and *ape* packages in R to extract the bat phylogeny from the Open Tree of Life and calculate branch lengths with Grafen’s method (Paradis *et al.*, 2004; Michonneau *et al.*, 2016). We first fit a null PGLS model (intercept only) using the *nlme* package to estimate phylogenetic signal as Pagel’s λ (Pagel, 1999). We next fit a PGLS model with bat family as the predictor to assess broad taxonomic patterns in hair cortisol. We then fit 15 PGLS univariate models with, dietary guild, foraging style, roost durability, fecundity, lifespan and colony size as predictors. We also fit five multivariate PGLS models including: BMR + body mass, dietary guild + fecundity, dietary guild + % invertebrates, dietary guild + lifespan + fecundity and dietary guild + fecundity + colony. We compared PGLS models with Akaike information criterion corrected for small sample sizes (AICc) and assessed fit with an adjusted R^2^ (Burnham and Anderson, 2002). All PGLS models included weighting by sampling variance to account for variable sample sizes per species (Pennell, 2015).

We used generalized linear models (GLMs) to determine which individual- and habitat-level factors influence hair cortisol for each bat species. We first evaluated the relationship between body mass and hair cortisol separately for each species. Next, we ran species-specific GLMs including sex, reproductive stage (by sex) and site disturbance as predictors. Not all covariates were tested for all species due to sample size restrictions. Total sample size and balanced sample sizes among levels were considered to select the number of covariates to include in the model for each species. We included disturbance in GLMs only for species present in more than one site (*Pteronotus mesoamericanus*, *P. mexicanus*, *Mactotus waterhousii*, *T. brasiliensis*, *Glossophaga soricina*, *D. rotundus*) since disturbance was treated as constant within sites. The only genus sampled in both Belize and Mexico was *Pteronotus*. The two species *P. mesoamericanus* (Belize) and *P. mexicanus* (Mexico) represent lineages considered conspecific until a few years ago, but are now thought to represent distinct species that diverged very recently based on molecular and morphometric evidence (Pavan and Marroig, 2016). Because their phenotypes and ecology are still very similar, we treated these as conspecific to test if there were differences in hair cortisol between representatives from the two regions (Mexico and Belize). Tukey post hoc tests were conducted for significant covariates. We compared effect sizes across bat species by evaluating the degree of overlap in 95% confidence interval for each GLM coefficient. All analyses used the natural logarithm of hair cortisol as the response variable and assumed Gaussian errors. We confirmed that all models fulfilled assumptions of normality, homoscedasticity and non-multicollinearity (variance inflation factors < 3). We report data as mean ± SD, unless otherwise noted.

## Results

### Ecological and evolutionary predictors of hair cortisol

**Figure 2:**
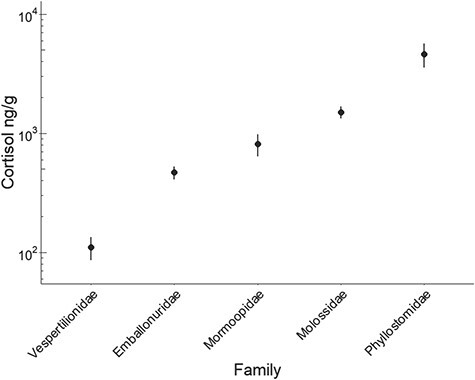
Mean and standard error of cortisol concentration in hair samples from 18 Neotropical bat species grouped by family. Summary statistics are displayed without adjusting for bat phylogeny. The y-axis is displayed on a log_10_ scale.

**Figure 3:**
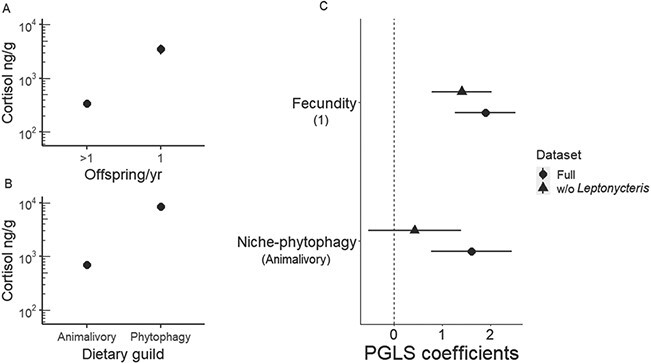
Left: Cortisol concentration in hair samples from 18 Neotropical bat species according to diet and annual fecundity. Y-axes are displayed on a log_10_ scale. A. Mean hair cortisol by number of offspring per year. B. Hair cortisol by dietary guilds (animalivory or phytophagy). C. Differences in parameter estimates for the PGLS model with and without *L. yerbabuenae.* Bars indicate the 95% confidence intervals. The reference values for each variable of the model are listed in parentheses.

We analysed 259 hair samples from 18 different bat species representing 5 families in Belize and Mexico (Table 1). Hair cortisol concentration across species varied by four orders of magnitude, ranging from 36.6 ± 40.5 ng/g in *Eptesicus furinalis* to 24 614 ± 14 780 ng/g in *L. yerbabuenae* (Table 1). Even though mean hair cortisol apparently differed among families (F_4,252 =_ 18.89; *P* < 0.01; *R*^2^ = 0.23; Fig. 2), this effect did not hold after accounting for phylogenetic relatedness (F_4,13_ = 1.84; *P* = 0.18; *R*^2^ = 0.16). Accordingly, we did not find strong phylogenetic signal in cortisol (Pagel λ = 0). Ecological and life history traits were instead better predictors of species-level cortisol levels (Table 2). Mean hair cortisol was best predicted by a model including both dietary guild and fecundity; however, only fecundity had a significant effect (F_2,15_ = 5.51; *P* = 0.01; *R*^2^ = 0.34; Table 2). Annual fecundity explained 24% of the variance in Neotropical bat mean hair cortisol. Species reported to have more than one pup per year had significantly lower cortisol than bats having only one pup per year (F_1,16_ = 6.22; *P* = 0.02; Fig. 3A). While phytophagous bats seem to have higher levels of cortisol in hair than animalivorous bats, this difference is not significant when considering the phylogenetic relatedness (Fig. 3B). Other ecological traits including roost durability, foraging style, and colony size were uninformative (Table 2). As cortisol levels varied by sex for some species (see below), we reran our model comparison after calculating species-level means for males and females separately. However, the above aggregate species-level results held when analysing the sexes separately (Supplementary Tables 2-3).

**Table 2:** PGLS models predicting hair cortisol (ln transformed) in Neotropical bats. Models are ranked by ΔAICc with the number of coefficients (*k*), Akaike weights (*w*_i_) and the adjusted *R^2^*

Model structure	df	ΔAICc	*w_i_*	R^2^
Dietary guild + fecundity	2	0	0.412	0.34
Fecundity	4	1.08	0.247	0.24
Dietary guild+ fecundity+ foraging style	5	3.30	0.081	0.30
1 (intercept only)	6	4.12	0.054	0
Dietary guild + fecundity+ lifespan	1	4.38	0.048	0.46
Roost durability	2	5.64	0.025	0
Dietary guild	3	5.68	0.024	0.01
BMR+ body mass	3	6.00	0.021	0
Sample	2	6.39	0.017	0.33
Foraging style	2	6.67	0.015	0
Colony size	4	8.01	0.007	0.37
Dietary guild + invertebrate%	3	8.35	0.006	−0.03
BMR + foraging style	3	8.92	0.005	−0.08
Family	5	9.12	0.004	0.16
Foraging style + WAR	3	9.15	0.004	−0.1
Lifespan	4	10.54	0.002	−0.06

The lesser long-nosed bat (*L. yerbabuenae*) showed particularly high hair cortisol (24 614 ± 14 780 ng/g). Because the high values of this species could bias inter-species comparisons, we assessed the sensitivity of our top models by excluding *L. yerbabuenae*. In Fig. 3C, we show the coefficients from the top PGLS models with and without this species. In both cases, fecundity was the best species-level predictor of hair cortisol regardless of including *L. yerbabuenae*.

### Individual-level analyses of bat hair cortisol

**Figure 4:**
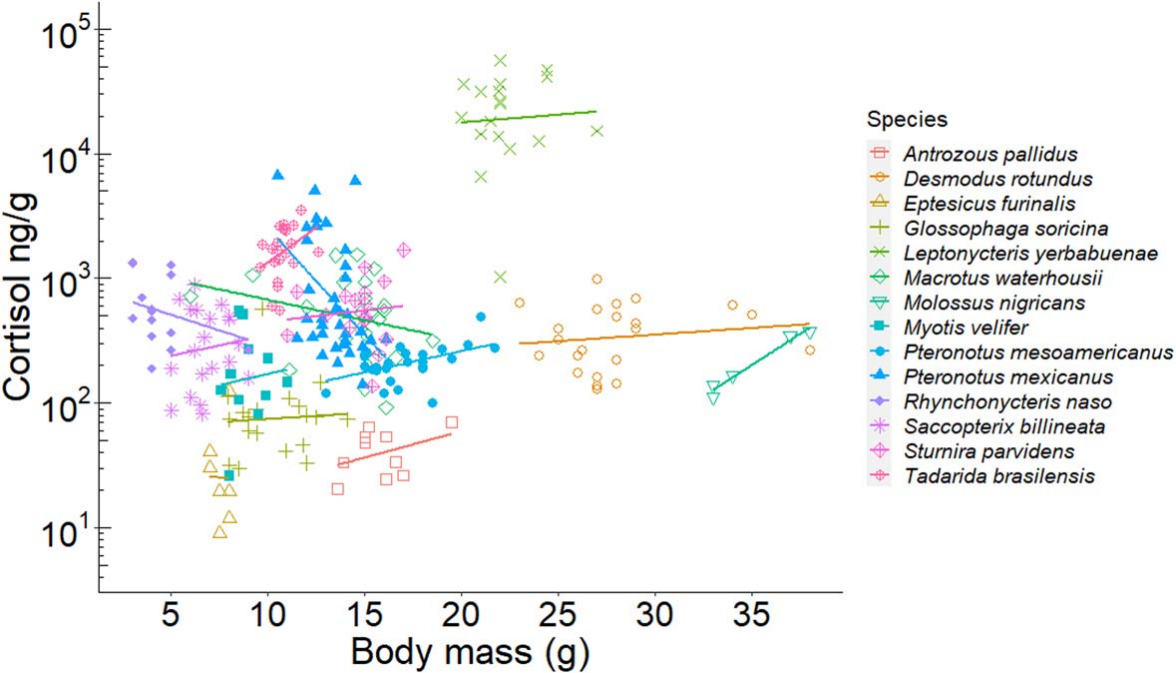
Relationship between hair cortisol concentration and body mass for each Neotropical bat species. Lines represent the GLM fit for each species. The y-axis is shown on a log_10_ scale.

**Figure 5:**
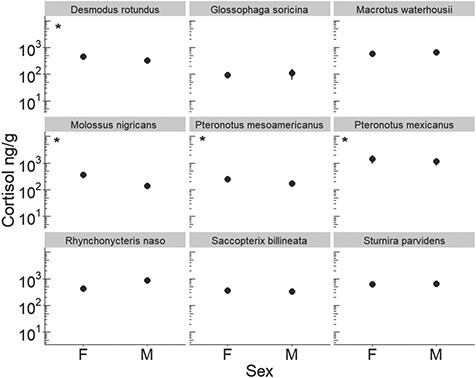
Mean and standard error for hair cortisol concentration by sex (F, female; M, male) for nine species of Neotropical bat species. Asterisks indicate species for which cortisol significantly varies by sex. Effects are only shown for species with balanced sample sizes per sex. Y-axes are shown on a log_10_ scale.

When investigating intra-specific variation, we found positive relationships between body mass and hair cortisol in two species: *P. mesoamericanus* (F_1,24_ = 7.34; *P* = 0.010; R^2^ = 0.23) and *Molossus nigricans* (F_1,4_ = 96.52; *P* < 0.01; *R*^2^ = 0.96; Fig. 4). The opposite trend was found in *P. mexicanus,* where heavier bats presented lower cortisol (F_1,33_ = 7.97; *P* < 0.01; *R*^2^ = 0.19). For *D. rotundus*, only sex was a significant predictor of hair cortisol (F_1,19_ = 4.39; *P* = 0.04; *R*^2^ = 0.19). Male vampire bats had significantly lower hair cortisol than females (t_20_ = 2.09; *P* = 0.02). Similarly, variation in hair cortisol in the moustached bat (*P. mesoamericanus*) and the mastiff bat (*M. nigricans*) was explained only by sex, with males having lower concentrations than females (t_4_ = 2.68; *P* = 0.01 and t_24_ = −6.373; *P* = 0.01, respectively; Fig. 5). When treating *P. mesoamericanus* (Belize) and *P. mexicanus* (Mexico) as one species, we found differences in hair cortisol between the two populations. Bats from Mexico had higher cortisol than their counterparts in Belize (F_1,59_ = 29.88; *P* < 0.01; *R*^2^ = 0.33). Within Mexico, hair cortisol in *P. mexicanus* was explained by site disturbance (F_2,31_ = 72.35; *P* < 0.001): bats roosting in Don Pancho cave (San Agustin island), a site with moderate disturbance (HMI = 0.38), showed significantly higher hair cortisol than bats roosting in El Salitre and La Fabrica caves in Colima (t_20_ = 9.94, *P* < 0.01; t_21_ = −10.29; Fig. 1). There was no effect of sex (t_33_ = −1.15; *P* > 0.31) or females’ reproductive stage (F_4,14_ 3.26; *P* = 0.06) on hair cortisol in *P. mexicanus*. For other species such as *E. furinalis* (F_1,12_ = 2.451; *P* = 0.64), *L. yerbabuenae* (F_1,17_ = 0.52; *P* = 0.94), *Saccopteryx billineata* (F_2,17_ = 0.18; *P* = 0.83), *Rhynchonycteris naso* (F_2,11_ = 2.60; *P* = 0.12), *G. soricina* (F_3,15_ = 0.13; *P* = 0.94), *Macrotus waterhousii* (F_2,18_ = 0.24; *P* = 0.78), *Sturnira parvidens* (F_2,14_ = 0.2052, *P* = 0.81) and *A. pallidus* (F_2,9_ = 0.506; *P* = 0.68), none of the individual- or habitat-level traits examined were informative predictors of hair cortisol levels.

## Discussion

Analysis of hair cortisol has become a popular method to study long-term stress in wild animals, offering several practical advantages (e.g. minimally invasive collection, easy sample storage and transport). An accurate interpretation of cortisol levels attributed to stress, however, requires a good understanding of the intrinsic and extrinsic drivers of baseline variation. Factors influencing hair cortisol in bats must be identified before hair cortisol can be used as a conservation tool to assess effects of environmental conditions on bat population health. In this study, we present the first quantification of hair cortisol in bats and describe relationships between hair cortisol levels and both intrinsic and ecological traits. Cortisol in blood, faeces and hair are known to be highly correlated in various mammals (e.g. chimpanzees, chipmunks and mice; Kalliokoski *et al.*, 2019). Therefore, although the concentration values are not directly comparable across different matrices, the effects of covariates can still be compared with our results.

One of the advantages of the use of hair as a physiological biomarker of stress is the longer window of time that it provides compared to other samples like blood and faeces. This feature, however, is only useful if the rate of hair growth and moulting patterns are known for the species of concern. Moulting cycles in bats are understudied, especially in tropical bats (Fraser *et al.*, 2013). Therefore, we cannot be certain of the precise temporal window that this biomarker could represent for our study species. While moult patterns for most of our tropical study species are unknown, we know that many temperate species have a one annual summer/fall moult cycle (Fraser *et al.*, 2013). If we assume our study species have a similar cycle, then the hair cortisol levels detected in our study would be reflecting primarily the circulating cortisol levels during one to two months of rapid hair growth between July and September. Moult in tropical bats, however, might not be confined to a distinct or single annual event, given the relative stable environmental conditions the tropics offer. Moult patterns in tropical bats therefore should be investigated further to allow inferences about the window of time over which cortisol levels in hair integrate and reflect circulating levels. Other aspects associated with differences in the timing of new fur growth, and therefore cortisol deposition in hair, could be related to local environmental variables (e.g. precipitation, temperature and food seasonality; Tiunov and Makarikova, 2007), as well as species–specific life history traits (e.g. reproduction and migration; Heydon *et al.*, 1995).

Overall, we found particularly high hair cortisol in most bat species compared to levels reported in hair for other small mammals (e.g. chipmunk [66-110 g] = 40.27–260.22 ng/g of hair; Mastromonaco *et al.*, 2014). Previous studies in bats, examining plasma and faeces, have also reported higher cortisol levels relative to similar samples from other mammal species (Lewanzik *et al.*, 2012; Kelm *et al.*, 2016; Hald, 2019). Some of the exceptional life history traits of bats, such as long lifespan and low fecundity, could explain why bats exhibit higher levels of GCs compared to other mammals (Austad and Fischer, 1991). According to life history theory, long-lived species with low reproduction rates are expected to prioritize their adult survival (i.e. future offspring) over current reproduction (Stearns, 1992), which could in turn favour higher investment in self-maintenance that might be facilitated by high baseline levels of GCs (Ricklefs and Wikelski, 2002).

### Ecological factors among species

Despite the similarity in the HPA hormonal pathways across vertebrates, baseline and stress-induced GC levels are context and species specific (Romero, 2004; French *et al.*, 2008; Crespi *et al.*, 2013; Kalliokoski *et al.*, 2019). In light of this, it is not surprising that hair cortisol levels in bats showed broad interspecific variation. The differences found could not be explained solely by taxonomic family or phylogenetic relatedness (λ = 0), which suggests that other environmental and ecological factors are influencing hair cortisol in Neotropical bats.

Among all the ecological traits evaluated, annual fecundity was the best predictor of hair cortisol. Species with lower fecundity showed higher concentrations of cortisol in hair. This relationship can be supported from a physiological perspective, considering that GCs play an important role modulating the production of reproductive hormones upstream in the hypothalamic–pituitary–gonadal axis, both under homeostatic and challenging conditions. This interaction, however, has only been clearly demonstrated within populations (e.g. individuals with high levels of cortisol reduce reproduction, reallocating energy current needs). It is unknown if interspecific variation in GCs is driven by the same mechanisms. Looking to other taxa, studies on birds have found similar results to those here in Neotropical bats, where avian species with low clutch size and few breeding events showed higher circulating GCs (Bókony *et al.*, 2009; Ouyang *et al.*, 2011). This pattern is supported by predictions from life history theory; for species with lower fecundity, the value of each offspring is higher than in species with relatively high fecundity (Lendvai *et al.*, 2007). Parents of more valuable broods would be predicted to be more ‘willing’ to invest in offspring survival, which might be facilitated by high baseline GC levels (Bókony *et al.*, 2009). Confirming if this mechanism explains the GCs levels found in our bat species would require more information about reproductive strategies (e.g. monoestry and polyoestry) and seasonality (Wingfield and Sapolsky, 2003), which is limited for most Neotropical species. A more practical rationale for our results could be related to differences in hair cortisol deposition among species with different reproductive cycles. For many species, pelage moulting occurs after breeding (Constantine, 1957; Dwyer, 1963; Cryan *et al.*, 2004; Measor *et al.*, 2017), given that both reproduction and hair growth are energetically demanding processes (Ling, 1970). Based on this pattern, we could expect that cortisol deposition in hair may reflect differences in breeding events among bat species.

Although diet explained additional variation in Neotropical bat hair cortisol, this variable was uninformative when considering phylogenetic relationships; hair cortisol did not vary significantly between our simplified dietary guilds. Further studies using a more accurate classification of diet (e.g. using stable isotopes or metabarcoding; Oelbaum *et al.*, 2019; Ingala *et al.*, 2021) could give more conclusive insights into the links between feeding strategies and cortisol levels in bats.

GCs play a key role in metabolic function, facilitating fuel mobilization (e.g. glucose, fatty acids) under normal and challenging conditions (Kuo *et al.*, 2015). A positive relationship between resting metabolic rate (RMR) and plasma cortisol levels has been reported for various mammalian species (including four species of bats), and this relationship has been suggested as a general pattern for mammals (Haase *et al.*, 2016). Due to the limited data on RMR for our study species, we used BMR as an indicator of energy expenditure. Different to what we expected, BMR was not an informative factor for cortisol variation in hair among the bats in our study. The positive relationship between cortisol levels and metabolic rate previously found in plasma might be obscured in studies of hair cortisol like ours, due to confounding factors such as moulting cycles and cortisol deposition rate. In addition, obtaining accurate BMRs in wildlife species (particularly free-ranging animals) is challenging, which raises questions about the quality of BMR data, especially in comparative studies (Genoud *et al.*, 2018). For future studies, a more realistic and informative indicator of energy turnover in free-ranging animals is the Daily Energy Expenditure (Speakman, 1997), which integrates the energy allocated in different activities such as foraging, commuting and thermoregulation (Butler *et al.*, 2004).

The relationship between body condition and GC release has been widely evaluated, because weight loss is one of the early responses to long-term stress in many species (Kitaysky *et al.*, 1999; Angelier *et al.*, 2009; Dickens and Romero, 2013). However, the direction of the effect of body condition on cortisol is context and species dependent (Crespi *et al.*, 2013). We used body mass as an indicator of body condition because it is a more informative metric than other indices in bats (McGuire *et al.*, 2018). We found different directions of the effect of body mass on hair cortisol. For two of the studied species (*M. nigricans* and *P. mesoamericanus*), heavier individuals showed higher concentrations of hair cortisol. In contrast, *P. mexicanus* showed a negative relationship between body mass and cortisol. These divergent results suggest that the relationship between cortisol and body condition of bats is not generally predictable and might be species specific. Hair cortisol only reflects the time window of fur growth. Therefore, it is possible that body condition might have changed since the individual’s last moult.

One species that stood out for its particularly high levels of cortisol was *L. yerbabuenae*. This species is highly mobile and migratory (Horner *et al.*, 1998; Buecher and Sidner, 2013; Medellin *et al.*, 2018). Migration itself was not considered in our analyses, because the degree to which bats may migrate seasonally is unclear for many of the species in our sample. Migratory behaviour, however, could explain such high cortisol concentrations in *L. yerbabuenae*. La Fábrica caves in Colima, one of our field sites, is known to be one of the starting points of the annual migration of *L. yerbabuenae* (Medellin *et al.*, 2018). The role of GCs during migration has been widely studied in birds, fish and some large mammals, but not in bats (Holberton, 1999; Romero, 2002; Wada, 2008). We hypothesize that premigratory fattening could explain the high hair cortisol levels observed in *L. yerbabuenae*, and we encourage future studies to address this question.

Consistent with other studies, we found differences in hair cortisol levels between sexes, albeit for only 4 of our 18 studied species: *D. rotundus*, *M. nigricans*, *P. mexicanus* and *P. mesoamericanus*. For these species, females showed higher cortisol than males, a trend that appears to hold for many mammalian species (Bechshøft *et al.*, 2011; Hau *et al.*, 2016; Rakotoniaina *et al.*, 2017; Dettmer *et al.*, 2018). Higher levels of GCs in females can be attributed to sex differences in the HPA axis activity, which are mainly mediated by gonadal steroid hormones (i.e. androgens and estrogens). For example, estradiol, which is more abundant in females than males, can enhance cortisol release, while androgens tend to reduce its production (Handa *et al.*, 1994). Other mechanisms underlying sex differences in HPA axis regulation and stress-related behaviours in mammals are reviewed by Zuloaga *et al.* (2020). Females have also shown differences in HPA axis activity depending on their life history stage, with GCs being higher during the late stages of pregnancy (Reeder *et al.*, 2004). Studies in a fruit-eating bat (*Artibeus jamaicensis*) and little brown myotis (*Myotis lucifugus)* have reported higher levels of plasma GCs in pregnant females (Reeder and Kramer, 2005; Klose *et al.*, 2006). Contrary to those findings, we did not find reproductive state to influence hair cortisol in our female-only model (i.e. for *P. mesoamericanus* in Belize). However, it may have been difficult to detect an effect, given the low number of pregnant females in our sample (*n* = 5, 24%) and the fact that moulting might not occur in conjunction with mating. Although cortisol has been proposed as the primary GC in bats (reviewed by Kwiecinski and Damassa, 2000), corticosterone is also detectable in circulation and has been identified to play an important role in reproduction (Koren *et al.*, 2012). Therefore, some effects of ecological traits could go unnoticed and the complexity of the stress response in bats could be oversimplified by quantifying only cortisol.

Bats have been proposed as good indicators of habitat quality due to their ecological diversity, wide distribution and potential sensitivity to disturbance (Jones *et al.*, 2009a; Cunto and Bernard, 2012; Stahlschmidt and Brühl, 2012). However, a clear correlation between environmental disturbance and cortisol levels, in faeces and blood, has not been reported in bats (Wada *et al.*, 2010; Allen *et al.*, 2011; Kelm *et al.*, 2016). Cortisol in hair could reflect better the effects of chronic stressors such as human settlements, and it is not sensitive to capture stress. We compared hair cortisol in three of our study species found in sites with varying fragmentation and agricultural activities in Mexico. We found an effect of disturbance in only one of these species, *P. mexicanus*, for which bats roosting in Don Pancho Cave island, a site with moderate disturbance, showed the highest concentrations of cortisol (Fig. 1). We speculate that the high levels found in this population could reflect differences in the cave microhabitat compared to the other caves in our Mexican sample. Don Pancho Cave is a narrow crevice estimated to have a higher colony size (100 000 individuals from 6 species; Téllez *et al.*, 2018) than the other sampled caves El Salitre (~10 000 individuals from 10 species; Torres-Flores *et al.*, 2012) and La Fábrica (>5000 individuals from four species). The high density of bats in the Don Pancho cave may increase agonistic social interactions (Creel *et al.*, 2013) and parasite transmission (Langwig *et al.*, 2012; Postawa and Szubert-Kruszyńska, 2014), factors that have been shown to increase cortisol levels in other mammals.

Physiological responses to chronic stress in wildlife are difficult to unravel and predict unless multiple responses at different levels of biological organization are evaluated simultaneously (Dickens and Romero, 2013). Hair cortisol offers great potential as a tool to monitor health in wild populations, particularly those already identified at risk (Kalliokoski *et al.*, 2019). For instance, chronically elevated cortisol levels have been linked to greater susceptibility to infection and disease severity (Davy *et al.*, 2017). Periodic surveys of hair cortisol could therefore help identify periods when bats might be more vulnerable to infection (e.g. white nose syndrome). Further, such surveys might also inform when individuals are more likely to shed zoonotic pathogens (e.g. henipaviruses and filoviruses; Plowright *et al.*, 2008; Davy *et al.*, 2017; McMichael *et al.*, 2017; Kessler *et al.*, 2018).

## Conclusions

The current study reports cortisol levels in hair of 18 Neotropical bat species from two countries and serves as a reference for future research using this method in wild bat populations. We found that fecundity and potentially diet are important ecological traits explaining interspecific variation in bat hair cortisol. Within species, female bats exhibited higher cortisol than males and the effect of body mass varied among species. Other factors that may be important at the individual level, such as parasite load and colony size, should be considered in future studies to have a more complete understanding of sources of variation on baseline GC levels within species. Importantly, studies looking at hair growth rate and moulting cycles in Neotropical bat species are imperative to give an accurate interpretation of hair cortisol as a biomarker of stress response. Applied properly, hair cortisol quantification is a powerful minimally invasive technique with multiple potential applications in bat ecology, physiology and conservation. Our findings and ongoing work will help to validate and apply hair cortisol as a monitoring tool in wild bat populations.

## Funding

This work was supported by the Natural Sciences and Engineering Research Council of Canada Discovery Grant (to K.C.W.), the Toronto Zoo Foundation (to G.M.), the ARCS Foundation and the American Museum of Natural History Theodore Roosevelt Memorial Fund (to D.J.B.) and the Taxonomic Mammalogy Fund of the American Museum of Natural History (to N.B.S.).

## Author contributions statement:

N.S.H. conceived the study and collected the samples. G.F.M. analysed the samples. N.S.H. and D.J.B. analysed the data and wrote the manuscript. N.B.S. provided support for collection and export permits and coordinated filed work logistics in Belize. K.C.W. Jr. secured funding for field and laboratory work. All authors reviewed drafts of the manuscript.

## Supplementary Material

Supplementary_material_CONPHYS-2020-150_coab053
