# Supplementary material for: Inter- and intra-specific variation in hair cortisol concentrations of Neotropical bats
Source: Conserv Physiol. 2021 Jul 14;9(1):coab053. doi: 10.1093/conphys/coab053 (PMC8278960; doi:10.1093/conphys/coab053)
Supplement: Supplementary_material_CONPHYS-2020-150_coab053 [file supplementary_material_conphys-2020-150_coab053.docx]

**Supplementary data:**

**Inter- and intra-specific variation in hair cortisol concentrations of Neotropical bats**

1. **Hair cortisol quantification validation**

**Bat hair recoveries**

Figure S1. *Eptesicus fuscus* hair Cortisol (p<0.001); recovery = 87.1% ± 3.5 [mean ± SE]

Figure S2. *Tadarida* *brasiliensis* hair Cortisol (p<0.01); recovery = 98.8% ± 6.3 [mean ± SE]

Figure S3. *Pteronotus mexicanus* hair Cortisol (p<0.001); recovery = 105.4% ± 4.7 [mean ± SE]

Figure S4. *Leptonycteris* *yerbabuena* hair Cortisol (p<0.001); recovery = 101.3% ± 5.2 [mean ± SE]

**Bat hair parallelisms**

Figure S5. *Eptesicus fuscus* hair Cortisol (p<0.001).

Figure S6. *Tadarida* *brasiliensis* hair Cortisol (p<0.01).

Figure S7. *Pteronotus mexicanus* hair Cortisol (p<0.001).

Figure S8*. Leptonycteris* *yerbabuena* hair Cortisol (p<0.001).

1. **Basal Metabolic Rate (BMR)**

Table 1. Data from Basal Metabolic Rate (BMR) and Relative basal metabolic rate (RBMR) for 18 Neotropical bat species extracted from the literature.

| **Species** | **Body mass (g)**  **(mean** ± SD**)** | | | **BMR**  **(mlO_2_/h)** | **RBMR**  (mlO_2_/hr.g | **Source** |
| --- | --- | --- | --- | --- | --- | --- |
| *Antrozous pallidus* | 15.8 | ± | 1.7 | 21.2 | 1.4 | (Licht and Leitner, 1967) |
| *Desmodus rotundus* | 28.0 | ± | 3.5 | 26.8 | 1.0 | (Mcnab, 1986) |
| *Eptesicus furinalis* | 7.6 | ± | 0.4 | 16.9 | 2.2 | (Willis *et al.*, 2005) |
| *Glossophaga soricina* | 10.3 | ± | 1.9 | 21.6 | 2.2 | (McNab, 1969; Mcnab, 1986) |
| *Lasiurus ega* | 12.0 | ± | 4.2 | 17.45 | 1.6 | (Genoud, 1993) |
| *Leptonycteris yerbabuenae* | 22.3 | ± | 1.7 | 36.5 | 1.6 | (Carpenter and Graham, 1967) |
| *Lophostoma evotis* | 19.0 | ± |  | 28.3 | 1.5 | Calculated |
| *Macrotus waterhousii* | 14.7 | ± | 1.9 | 14.6 | 1.0 | Bell *et al.* (1986) |
| *Mimon cozumelae* | 25.0 | ± | 0.0 | 32.5 | 1.3 | Calculated |
| *Molossus rufus* | 35.0 | ± | 2.3 | 42 | 1.2 | (Mcnab, 1986) |
| *Myotis velifer* | 9.0 | ± | 1.0 | 12.6 | 1.4 | (Riedesel and Williams, 1976) |
| *Pteronotus mesoamericanus* | 17.1 | ± | 2.1 | 30.7 | 1.8 | (Bonaccorso *et al.*, 1992) |
| *Pteronotus parnellii* | 13.4 | ± | 1.2 | 30.7 | 2.3 | (Bonaccorso et al. 1992) |
| *Rhynchonycteris naso* | 4.5 | ± | 1.5 | 16.2 | 3.8 | Baudinette *et al.* (2000) |
| *Saccopterix billineata* | 6.8 | ± | 1.1 | 14.5 | 2.2 | (Genoud and Bonaccorso, 1986) |
| *Sturnira parvidens* | 14.6 | ± | 1.5 | 34 | 2.4 | (Audet and Thomas, 1997) |
| *Tadarida brasilensis* | 10.7 | ± | 0.6 | 15.3 | 1.4 | (Licht and Leitner, 1967) |
| *Trachops cirrhosus* | 31.5 | ± | 0.5 | 38.2 | 1.2 | Calculated |

References

Audet D, Thomas DW (1997) Facultative hypothermia as a thermoregulatory strategy in the phyllostomid bats, Caroliia perspicillata and Sturnira lilium. *J Comp Physiol - B Biochem Syst Environ Physiol* 167: 146–152.

Bonaccorso FJ, Arends A, Genoud M, Cantoni D, Morton T (1992) Thermal ecology of moustached and ghost-faced bats (Mormoopidae) in Venezuela. *J Mammal* 73: 365–378.

Carpenter RE, Graham JB (1967) Physiological responses to temperature in the long-nosed bat, Leptonycteris sanborni. *Comp Biochem Physiol* 22: 709–722.

Genoud M (1993) Temperature regulation in subtropical tree bats. *Comp Biochem Physiol -- Part A Physiol* 104: 321–331.

Genoud M, Bonaccorso FJ (1986) Temperature Regulation, Rate of Metabolism, and Roost Temperature in the Greater White-Lined Bat Saccopteryx bilineata (Emballonuridae). *Physiol Zool* 59: 49–54.

Licht P, Leitner P (1967) Physiological responses to high environmental temperatures in three species of microchiropteran bats. *Comp Biochem Physiol* 22: 371–387.

Mcnab BK (1986) The Influence of Food Habits on the Energetics of Eutherian Mammals.

McNab BK (1969) The economics of temperature regulation in neutropical bats. *Comp Biochem Physiol* 31: 227–268.

Riedesel ML, Williams BA (1976) Continuous 24-hour oxygen consumption studies of Myotis velifer. *Comp Biochem Physiol -- Part A Physiol* 54: 95–99.

Willis CKR, Lane JE, Liknes ET, Swanson DL, Brigham RM (2005) Thermal energetics of female big brown bats (Eptesicus fuscus). doi:10.1139/Z05-074


Table 2. PGLS models predicting hair cortisol (ln transformed) in female bats. Models are ranked by ΔAICc with the number of coefficients (*k*), Akaike weights (*w*_i_), and the adjusted *R^2^*.

| **Model structure** | **df** | **ΔAICc** | ***w_i_*** | **R^2^** |
| --- | --- | --- | --- | --- |
| **~fecundity** | 2 | 0.00 | 0.53 | 0.3 |
| **~ dietary guild+ fecundity** | 3 | 3.00 | 0.12 | 0.25 |
| ~ 1 (intercept only) | 1 | 3.36 | 0.10 | 0 |
| ~ roost durability | 2 | 5.42 | 0.04 | -0.03 |
| ~BMR+ body mass | 2 | 5.64 | 0.03 | -0.04 |
| ~sample | 2 | 5.84 | 0.03 | -0.05 |
| ~dietary guild | 2 | 5.88 | 0.03 | -0.05 |
| ~foraging style | 2 | 6.09 | 0.02 | -0.08 |
| ~foraging style + WAR | 3 | 6.33 | 0.02 | 0.08 |
| ~ dietary guild+ fecundity+ foraging style | 4 | 6.83 | 0.02 | 0.19 |
| ~family | 5 | 6.96 | 0.02 | 0.37 |
| ~dietary guild + invertebrate% | 3 | 8.41 | 0.01 | -0.09 |
| ~BMR+ body mass | 3 | 8.42 | 0.01 | -0.09 |
| ~BMR + foraging style | 3 | 8.94 | 0.01 | -0.14 |
| ~ dietary guild+ foraging style | 3 | 9.13 | 0.01 | -0.15 |
| ~colony size | 4 | 11.96 | 0.00 | -0.25 |
| ~lifespan | 4 | 13.41 | 0.00 | -0.29 |
| ~ dietary guild + fecundity+ lifespan | 6 | 17.35 | 0.00 | 0.06 |

Table 3. PGLS models predicting hair cortisol (ln transformed) in male bats. Models are ranked by ΔAICc with the number of coefficients (*k*), Akaike weights (*w*_i_), and the adjusted *R^2^*.

| **Model structure** | **df** | **ΔAICc** | ***w_i_*** | **R^2^** |
| --- | --- | --- | --- | --- |
| **~ dietary guild+ fecundity** | 3 | 0.00 | 0.42 | 0.35 |
| **~ fecundity** | 2 | 1.48 | 0.20 | 0.22 |
| **~ dietary guild+ fecundity+ foraging style** | 4 | 2.75 | 0.11 | 0.3298 |
| ~ dietary guild + fecundity+ lifespan | 6 | 3.96 | 0.06 | 0.48 |
| ~ 1 (intercept only) | 1 | 4.37 | 0.05 | 0 |
| ~sample | 2 | 4.57 | 0.04 | 0.06 |
| ~dietary guild | 2 | 5.64 | 0.03 | 0.02 |
| ~ roost durability | 2 | 6.00 | 0.02 | -0.01 |
| ~BMR | 2 | 6.42 | 0.02 | -0.03 |
| ~foraging style | 2 | 6.82 | 0.01 | -0.05 |
| ~ dietary guild+ foraging style | 3 | 8.06 | 0.01 | -0.02 |
| ~dietary guild + invertebrate% | 3 | 8.48 | 0.01 | -0.03 |
| ~BMR + foraging style | 3 | 9.22 | 0.00 | -0.09 |
| ~BMR+ body mass | 3 | 9.30 | 0.00 | -0.09 |
| ~foraging style + WAR | 3 | 9.65 | 0.00 | -0.11 |
| ~colony size | 4 | 9.72 | 0.00 | 0.12 |
| ~family | 5 | 10.03 | 0.00 | 0.13 |
| ~lifespan | 4 | 10.78 | 0.00 | -0.06 |
